# PIFA-N Multifactor Model to Predict Adverse Outcomes for Chronic Heart Failure Patients

**DOI:** 10.14740/jocmr6518

**Published:** 2026-04-15

**Authors:** Natalia A. Dragomiretskaya, Anastasia V. Tolmacheva, Aida I. Tarzimanova, Anna E. Pokrovskaya, Tatiana S. Vargina, Tatiana A. Safronova, Ivan D. Medvedev, Antonina A. Abramova, Daria D. Vanina, Valery I. Podzolkov

**Affiliations:** a Department of Internal Medicine No. 2, Institute of Clinical Medicine, Federal State Autonomous Educational Institution of Higher Education I.M. Sechenov First Moscow State Medical University of the Ministry of Health of the Russian Federation (Sechenovskiy University), Moscow, Russian Federation

**Keywords:** Chronic heart failure, Multifactor prognostic model, Prognostic factors, NT-proBNP

## Abstract

**Background:**

In recent years, special attention has been given to developing multifactor models that support a more personal approach to assessing prognosis for chronic heart failure (CHF) patients with varying degrees of systolic dysfunction. The existing scales, namely the Seattle Heart Failure Model (SHFM), Meta-Analysis Global Group in Chronic HF (MAGGIC-HF), PARADIGM Risk of Events and Death in the Contemporary Treatment (PREDICT-HF), and the Barcelona Bio-HF (BCN-Bio-HF) risk calculator, contain numerous variables and biomarkers not readily accessible in actual clinical practice and thus fall short of meeting physicians’ requirements. The study aimed to develop a multifactor model for assessing the risk of comorbid CHF patients developing adverse outcomes.

**Methods:**

Our study included 233 patients (129 male and 104 female) aged 73.2 ± 12.4 years, admitted to the Internal Medicine Clinic of the Sechenov University Clinical Hospital No. 4 with symptoms of New York Heart Association Functional Classification (NYHA FC) II to IV CHF, including 91 patients with heart failure with preserved ejection fraction (CHFpEF), 69 with heart failure with mildly reduced ejection fraction (CHFmrEF), and 63 with heart failure with reduced ejection fraction (CHFrEF). All the patients had given their informed and written consent to take part in the study. They were given a standard clinical examination and additionally tested for N-terminal pro-B-type natriuretic peptide (NT-proBNP), soluble interleukin-like protein receptor (sST2), galectin-3, hepcidin, and copeptin levels. Prospective follow-up lasted for 36 ± 3 months. The primary endpoint was defined as death from all causes. The findings were processed statistically using Statistica 12.0 and SPSS software.

**Results:**

All-cause mortality was 33.1% in the cohort examined. We used uni- and multivariate regression analysis to develop a PIFA-N prognostic mathematical model of adverse outcome that factored in community-acquired pneumonia diagnosed on inclusion in the study (odds ratio (OR): 3.09, 95% confidence interval (CI) 1.13–8.5, P = 0.028), a previous myocardial infarction (OR: 4.26, 95% CI 1.54–11.7, P = 0.005), presence of any form of atrial fibrillation (OR: 3.13, 95% CI 1.05–9.2, P = 0.039) and/or anemia (OR: 3.18, 95% CI 1.003–10.1, P = 0.049), and NT-proBNP level (OR: 1.0005, 95% CI 1.0002–1.0008, P = 0.002). Other biomarkers studied showed no predictive value. Our PIFA-N model is 77.1% sensitive and 77.3% specific (area under receiver operating characteristic curve (ROC AUC) 0.845), which meets high efficiency criteria.

**Conclusion:**

We analyzed CHF patients’ 3-year survival rate to develop an efficient prognostic model for assessing the risk of lethal outcome, whose arguments are easy-to-check conditions and routine laboratory test figures that can be established without any sophisticated examination techniques.

## Introduction

Chronic heart failure (CHF) remains a topical issue faced by the worldwide medical community [1, 2]. Cardiovascular diseases are absolute leaders among all causes of death in developed countries and the Russian Federation alike [3, 4]. CHF is the expectable final stage of most cardiovascular diseases that contributes up to 40% to overall cardiovascular mortality [1, 4]. In the years to come, the number of CHF patients is expected to grow due to general population aging, an increasing burden of cardiovascular diseases, and growing numbers of younger CHF patients with preserved ejection fraction who suffer from arterial hypertension, obesity, and metabolic disorders [5]. Despite the appearance of new information about CHF pathogenesis and improved methods of diagnosis and treatment, the patients’ prognosis remains poor, no matter whether their CHF features a preserved, mildly reduced, or reduced ejection fraction [1, 2].

In the recent years, special attention has been given to developing multifactor models that help specify prognosis for CHF patients with various degrees of systolic dysfunction. A number of scales have been developed by now to assess the risk of adverse prognosis for СHF patients. The most frequently used ones are the Seattle Heart Failure Model (SHFM), the Meta-Analysis Global Group in Chronic HF (MAGGIC-HF), PARADIGM Risk of Events and Death in the Contemporary Treatment (PREDICT-HF), and the Barcelona Bio-HF (BCN-Bio-HF) risk calculator [6–8]. Those models’ shared difficulties are, firstly, the use of many variables (up to 20), with the resultant calculation time costs, and, secondly, that they factor in a number of biomarkers not routinely measured (highly sensitive troponin and sST2 tests for the BCN-Bio-HF calculator) [8]. A recent study by Codina et al (2021) compares those four risk scales and concludes that none shows a visible advantage over the rest [9]. Therefore, the search for convenient and accurate scales to assess CHF patients’ risk of adverse outcomes is still ongoing [9, 10].

The study aimed to develop a multifactor model for assessing the risk of CHF patients developing adverse outcomes across the whole left ventricular ejection fraction (LVEF) spectrum.

## Materials and Methods

### Study design

Our single-institution prospective study involved 233 patients (129 male and 104 female) aged 73.2 ± 12.4 years, admitted to the University Clinical Hospital No. 4 of the Sechenov University with symptoms of New York Heart Association Functional Classification (NYHA FC) II to IV CHF in 2019 and 2020. Of these, 91 patients were diagnosed with heart failure with preserved ejection fraction (CHFpEF), 69 with heart failure with mildly reduced ejection fraction (CHFmrEF), and 63 with heart failure with reduced ejection fraction (CHFrEF). Diagnosis of CHFpEF, CHFmrEF, or CHFrEF was established by the attending physicians based on the echocardiographic findings and clinical presentation according to the current European Society of Cardiology/American College of Cardiology (ESC/ACC) guidelines. The inclusion criteria were: CHF diagnosis verified on the basis of clinical and echocardiographic characteristics 6 months or longer before; N-terminal pro-B-type natriuretic peptide (NT-proBNP) level above 125 pg/mL.

The patient exclusion criteria were defined as grave diseases that could affect the clinical symptoms; levels of biochemical markers; course and prognosis of pathology such as acute and grave renal diseases with glomerular filtration rate by Chronic Kidney Disease Epidemiology Collaboration formula (GFR_CKD-EPI_) < 15 mL/min/1.73 m^2^ or presence of Kidney Disease: Improving Global Outcomes (KDIGO) 2020 criteria of acute renal damage; primary hepatic pathology (of viral, toxic or other established etiology); acute coronary syndrome; cardiac surgery or revascularizing procedures in the preceding 6 months; an acute cerebrovascular accident in the preceding 6 months; acute or chronic autoimmune or infectious inflammatory diseases (except community-acquired congestive pneumonia); severe anemia; type 1 diabetes mellitus; cancers and hematological malignancies; and decompensated thyroid dysfunction. The Charlson Comorbidity Index was calculated by the standard algorithm that factors in 19 nosological units, ranked by their degree of influence on prognosis and assigned weighting factors between 1 and 6, and also the patients’ age (+1 point per 10 years of life after 40). Besides, all diseases with the weighting factors of 6 (malignancies with metastasis and acquired immune deficiency syndrome (AIDS)) and 3 (moderate to severe liver disease (with portal hypertension), and some diseases with the weighting factor of 2 (malignancies without metastasis, leukemia, and lymphoma) were criteria for exclusion from the study.

The patients were given a standard examination that included a complete blood count and blood chemistry test, standard electrocardiography (ECG), transthoracic echocardiogram, chest X-ray and, additionally, enzyme linked immunosorbent assay (ELISA) tests for the levels of biomarkers: NT-proBNP, galectin-3, soluble interleukin-like protein receptor (sST2), haptoglobin, hepcidin, and copeptin. The primary endpoint was defined as death from all causes. Follow-up lasted for 36 ± 3 months.

### Ethical approval

Our study was authorized by the Local Ethics Committee of the Sechenov University (Minutes No. 14-22 of 7 July 2020). We complied with all the provisions of the Helsinki Declaration and with the ethical principles of medical studies involving human subjects and Good Clinical Practice.

### Statistical data analysis

We used STATISTICA 12.0 (StatSoftInc, USA) and SPSS-16 (USA) software for statistical data analysis. The Kolmogorov-Smirnov and Shapiro-Wilk tests were used to establish normal distribution of quantitative indicators. To compare numeric data from two unrelated sets, we used the Mann-Whitney U-test. The data obtained are presented as the median (Me) and interquartile range (Q1; Q3). For frequency analysis, we used the Pearson χ^2^ test. With abnormally distributed attributes, we assessed correlation intensity using the Spearman correlation coefficient (r), and with normally distributed ones, the Pearson correlation coefficient (r). The survival rate was analyzed with Kaplan-Meier curves built and their divergence tested for significance using the Log-rank test. To assess patients’ prognosis, we used binominal logistic regression with odds ratio (OR) and 95% confidence interval (95% CI) calculation, which helps identify independent predictors of primary endpoint occurrence. Variations with Student’s coefficient P < 0.05 were considered statistically significant.

## Results

All-cause mortality in the cohort examined was 33.1% (77 patients). Cardiovascular mortality (33 patients, or 42.9%) was lower than mortality from other causes (44 patients, 57.1%). The most frequent cause of cardiovascular mortality among the patients was CHF decompensation (23 patients, 69.7%), acute (two patients, 6%) or recurrent (three patients, 9%) myocardial infarction, and acute cerebrovascular accident (three patients, 9%). One death was caused by popliteal artery thrombosis and moist gangrene. Another patient presented with signs of sudden cardiac death.

Pneumonia—predominantly caused by the SARS-CoV-2 virus—was the leading cause of non-cardiovascular mortality, accounting for 35 cases (84.6%) of such deaths.. Three patients (6.8%) developed cancer (pulmonary, gastric) or hematological malignancies (lymphoma compounded by autoimmune hemolytic anemia). One patient with grave CHF and 23% ejection fraction (EF) died of *Clostridium tetani* infection.

### Patients’ clinical profile

The patients who reached the endpoint were older, with more severe course of CHF, and a heavier burden of cardiac and non-cardiac comorbidities such as previous myocardial infarction, atrial fibrillation, and community-acquired pneumonia (Table 1).

**Table 1:** Comparative Analysis of the Clinical Characteristics and Backgrounds of Patients Who Reached/Did Not Reach the Endpoint

Indicator	Endpoint not reached (n = 146)	Endpoint reached (n = 77)	Р
Age, years, Me (Q1; Q3)	72 (64; 79.8)	78 (70; 82)	0.02
Male, n (%)	56 (38.4%)	28 (36.4%)	0.87
BMI, kg/m^2^, Me (Q1; Q3)	30.4 (26.5; 36.8)	30.6 (25.7; 34.8)	0.18
NYHA FC, mean ± SD	2.88 ± 0.65	3.2 ± 0.56	0.0005
Cardiac comorbidities			
Arterial hypertension, n (%)	139 (95.2%)	71 (97%)	0.25
SBP on admission, mm Hg, mean ± SD	137 ± 24.5	135 ± 22.3	0.41
Ischemic heart disease, n (%)	141 (96.6%)	69 (95%)	0.54
Post-infarction cardiosclerosis, n (%)	62 (42.5%)	48 (65.8%)	0.001
CABG history, n (%)	7 (4.7%)	6 (8%)	0.45
PCI history, n (%)	16 (11%)	9 (12%)	0.71
Rheumatic heart defect, n (%)	5 (3.4%)	6 (8%)	0.12
Degenerative heart defect, n (%)	20 (13.7%)	7 (9.6%)	0.35
Heart defect in outcome of infectious endocarditis, n (%)	2 (1.4%)	1 (1.3%)	0.76
Dilated cardiomyopathy, n (%)	8 (5.5%)	3 (4.3%)	0.73
Atrial fibrillation, n%	89 (61%)	55 (75%)	0.04
Pacemaker, n (%)	10 (6.8%)	7 (9.5%)	0.54
Non-cardiac comorbidities			
ACVA history, n (%)	11 (7.5%)	9 (12%)	0.23
Community-acquired pneumonia on admission, n (%)	42 (28.6%)	41 (56%)	0.0002
Bronchial obstructive diseases, n (%)	49 (33.5%)	25 (34%)	0.68
Pulmonary thromboembolism history, n (%)	3 (2%)	5 (7%)	0.12
Obstructive sleep apnea syndrome, n (%)	17 (11.6%)	4 (5%)	0.14
Mild anemia, n (%)	28 (19.2%)	17 (23%)	0.43
Type 2 DM, n (%)	51 (34.9%)	19 (26%)	0.23
HbA1c	6.2 (5.6; 6.9)	6.6 (5.9; 7.7)	0.19
Thyroid diseases, n (%)	32 (21.9%)	18 (25%)	0.67
Duodenal ulcer disease in remission/chronic gastroduodenitis, n (%)	46 (31.5%)	16 (22%)	0.14
Chronic kidney disease, n (%)	80 (54.7%)	52 (71%)	0.37
Joints and spine diseases, n (%)	32 (21.9%)	17 (22.3%)	0.63
Charlson Comorbidity Index, Me (Q1; Q3)	10.8 (9.6; 13.1)	12 (11; 13.8)	0.01

ACVA: acute cerebrovascular accident; BMI: body mass index; CABG: coronary artery bypass graft; DM: diabetes mellitus; HbA1c: glycated hemoglobin; Me (Q1; Q3): median (interquartile range); NYHA FC: New York Heart Association Functional Classification; PCI: percutaneous coronary intervention.

The patients who reached the endpoint were significantly older and had higher NYHA FC CHF; they had significantly more frequent myocardial infarction histories and pneumonia diagnoses on inclusion in the study, and significantly higher values of Charlson Comorbidity Index.

### Drug therapy

No differences in the medications administered were found between the groups (Table 2).

**Table 2:** Comparison of the Therapy Given to Patients Who Reached/Did Not Reach the Endpoint

Indicator	Endpoint not reached (n = 146)	Endpoint reached (n = 77)	Р
ACEIs (%)	110 (75.3%)	63 (81.8%)	0.512
ARBs, n (%)	22 (15.1%)	9 (11.6%)	0.313
Valsartan/sacubitryl, n (%)	5 (3.4%)	3 (3.9%)	0.897
Beta blockers, n (%)	151 (97%)	69 (95%)	0.541
MRAs, n (%)	72 (49.3%)	32 (41.5%)	0.412
Loop diuretics, n (%)	95 (65.1%)	66 (85.7%)	0.067
Thiazide and thiazide-like diuretics, n (%)	17 (11.6%)	8 (10.4%)	0.815
SGLT2Is, n (%)	51 (32.9%)	29 (37.7%)	0.614
Cardiac glycosides (digoxin), n (%)	12 (8.2%)	7 (9.1%)	0.733
Statins, n (%)	78 (53.4%)	52 (67.5%)	0.145
Amiodarone, n (%)	22 (15.1%)	18 (23.4%)	0.188
Sotalol, n (%)	9 (6.1%)	4 (5.2%)	0.765
Antiplatelet agents (aspirin), n (%)	25 (17.1%)	14 (18.1%)	0.855
DOACs, n (%)	85 (58.2%)	54 (70.1%)	0.072
Calcium antagonists, n (%)	7 (4.8%)	4 (5.2%)	0.814

ACEIs: angiotensin converting enzyme inhibitors; ARBs: angiotensin receptor blockers; DOACs: direct oral anticoagulants; MRAs: mineralocorticoid receptor antagonists; SGLT2Is: type 2 sodium-glucose cotransporter inhibitors.

### Echocardiographic examinations

The cohort of the patients who reached the endpoint presented with marked signs of maladaptive remodeling in the form of left ventricular and left atrial cavity dilation, significantly lower LVEF (P = 0.001), and significantly higher systolic pulmonary artery pressure (P < 0.004) (Table 3).

**Table 3:** Comparative Analysis of Echocardiographic Characteristics of CHF Patients Who Reached/Did Not Reach the Endpoint

Indicator	Endpoint not reached (n = 146)	Endpoint reached (n = 77)	Р
LVEF, %	50 (41; 59.8)	41 (36; 51.5)	0.001
LV EDD, mm	48 (43; 52)	50 (46; 52.2)	0.853
LV ESD, mm	35 (31; 40)	41 (31; 44)	0.320
LV EDV, mL	100 (83; 122)	119 (107; 152)	0.03
LV ESV, mL	42 (39; 71)	67 (43; 96)	0.001
LVSV, mL	64 (60; 72)	67 (54; 70)	0.568
IVS, mm	11.5 (9.8; 12.5)	12 (11; 12.5)	0.763
LVPW, mm	10 (9; 10.5)	10 (9; 10.5)	0.689
LVMI, g/m^2^	100 (81; 117)	110 (92; 126)	0.09
LA volume, mL	71 (66; 95)	90 (74; 101)	0.03
sPAP, mm Hg	28 (21; 44)	40 (26.5; 52)	0.004
ICVd, mm	20 (20; 21)	20.5 (19; 23)	0.129
RVEF, %	62 (57; 67)	57 (53; 63)	0.304

LVEF: left ventricular ejection fraction; LV EDD: left ventricular end-diastolic dimension; LV ESD: left ventricular end-systolic dimension; LV EDV: left ventricular end-diastolic volume; LV ESV: left ventricular end-systolic volume; LVSV: left ventricular stroke volume; IVS: interventricular septum thickness in diastole; LVPW: left ventricular posterior wall thickness in diastole; LVMI: left ventricular mass index; LA volume: left atrial volume; sPAP: systolic pulmonary artery pressure; ICVd: inferior vena cava diameter; RVEF: right ventricular ejection fraction.

### Laboratory values in the groups under review

Comparative analysis of most blood count figures showed no significant differences in the groups, while some indicators in the blood chemistry tests would differ significantly (Table 4).

**Table 4:** Comparative Analysis of Laboratory Characteristics of CHF Patients Who Reached/Did Not Reach the Endpoint

Indicator	Endpoint not reached (n = 146)	Endpoint reached (n = 77)	Р
Leukocytes, × 10^9^/L	6.92 (5.79; 8.59)	7.51 (5.99; 8.86)	0.25
Hemoglobin, g/L	139 (126; 148)	134 (121; 144)	0.06
Platelets, × 10^9^/L	220 (178; 262)	202 (164; 259)	0.58
ESR, mm/h	20.5 (11.8; 33)	20 (10.8; 38.8)	0.43
Creatinine, µmol/L	102 (92.3; 117)	114 (93.4; 138.4)	0.042
GFR_CKD-EPI_, mL/min/1.73 m^2^	53 (44.9; 64)	49 (36.9; 61)	0.07
Urea nitrogen, mmol/L	7.5 (5.9; 9.3)	9.5 (6.7; 12.7)	0.0001
Potassium, mmol/L	4.6 (4.4; 4.9)	4.6 (4.01; 5)	0.84
Sodium, mmol/L	144 (141; 147)	142 (138; 145)	0.21
Iron, µmol/L	12 (8.4; 17.1)	8.5 (6.3; 11.5)	0.34
AST, U/L	26 (21.3; 33)	28 (20; 39)	0.32
ALT, U/L	22 (17; 34)	20.2 (13.7; 26)	0.36
Gamma-glutamyl transpeptidase, U/mL	41 (25.5; 53.5)	33.5 (25.3; 41.8)	0.44
Alkaline phosphatase, U/mL	199 (155; 247)	230 (176; 298)	0.12
Total bilirubin, µmol/L	14.9 (10.6; 20.7)	15.1 (10.4; 21.9)	0.41
Direct bilirubin, µmol/L	4.9 (3.6; 6.4)	8.6 (5.7; 10.9)	0.07
Albumin, g/L	41.9 (36.5; 44.2)	38.1 (33.7; 40.1)	0.03
Total cholesterol, mmol/L	4.77 (3.7; 5.9)	4.01 (3.26; 5.07)	0.001
HDLs, mmol/L	1.25 (1.02; 1.54)	1.17 (0.81; 1.61)	0.39
LDLs, mmol/L	2.92 (2.05; 3.74)	2.34 (1.85; 2.7)	0.001
VLDLs, mmol/L	0.59 (0.42; 0.99)	0.53 (0.37; 0.71)	0.03
Glucose, mmol/L	6.04 (5.3; 7.2)	6.37 (5.3; 7.3)	0.83
INR	1.02 (0.94; 1.14)	1.15 (0.98; 1.38)	0.02
MELD-XI, points	10.6 (8.2; 12.8)	12.2 (9.7; 15.1)	0.001
NT-proBNP, pg/mL	468.1 (203.2; 1,142.4)	987 (421.7; 2,365)	0.002
Copeptin, ng/mL	6.96 (5.11; 8.23)	6.64 (5.33; 9.16)	0.629
ST2, ng/mL	23.29 (14.9; 33.1)	53.76 (21.1; 93.5)	0.017
Galectin-3, ng/mL	8.85 (6.67; 11.53)	9.92 (5.88; 10.69)	0.718
Haptoglobin, ng/mL	1086 (686; 1,717)	1286 (748.1; 1,957.9)	0.689
Hepcidin, ng/mL	27.1 (22.94; 53.72)	22.5 (19.09; 37.17)	0.207

ALT: alanine aminotransferase; AST: aspartate aminotransferase; ESR: erythrocyte sedimentation rate; GFR_CKD-EPI_: glomerular filtration rate estimated by CKD-EPI; HDLs: high-density lipoproteins; INR: international normalized ratio; LDLs: low-density lipoproteins; NT-proBNP: N-terminal pro-B-type natriuretic peptide; ST2: soluble interleukin-like protein receptor; VLDLs: very low-density lipoproteins.

The patients who reached the endpoint had more marked signs of renal and hepatic dysfunction: elevated levels of creatinine and urea, low levels of serum albumin, total cholesterol and its pro-atherogenic fractions, LDLs and VLDLs, while the proportion of patients on lipid-lowering therapy was comparable. Of all the biomarkers analyzed, only NT-proBNP and ST2 were veritably higher in the cohort of patients who reached the endpoint. No other marker levels were found to differ significantly.

### Poor prognosis risk factors for CHF patients

In the presence of numerous clinical, instrumental, and laboratory factors that could affect patients’ prognosis, we did univariate and multivariate regression analysis to assess our patients’ personal risk of unfortunate outcome (Table 5).

**Table 5:** Results of Uni- and Multivariate Cox Regression Analysis (Adjusted for Age, Sex, BMI, NYHA Class, and Renal Function (Creatinine/Urea)) for Assessing CHF Patients’ Lethal Outcome Risk

Risk factors	Univariate regression analysis			Multivariate regression analysis		
	RR	95% CI	P	OR	95% CI	P
Age	1.024	0.999–1.049	0.060			
Male gender	0.953	0.58–1.563	0.850			
BMI	0.969	0.936–1.004	0.083			
LVEF < 40%	2.451	1.478–4.064	0.001			
Community-acquired pneumonia	2.398	1.451–3.962	0.021	3.09	1.13–8.5	0.028
AMI history	4.262	1.68–11.78	< 0.001	4.26	1.54–11.7	0.005
Atrial fibrillation	3.134	1.06–9.24	0.034	3.13	1.05–9.2	0.039
Anemia	3.175	1.003–10.04	0.033	3.18	1.003–10.1	0.049
MELD-XI	2.345	1.274–4.315	0.006			
GFR_CKD-EPI_	0.991	0.975–1.007	0.246			
NT-proBNP	1.0004	1.0001–1.0005	0.002	1.0005	1.0002–1.0008	0.002
sST2	1.011	1.007–1.021	0.035			

AMI: acute myocardial infarction; BMI: body mass index; CI: confidence interval; GFR_CKD-EPI_: glomerular filtration rate estimated by CKD-EPI; LVEF: left ventricular ejection fraction; OR: odds ratio; NT-proBNP: N-terminal pro-B-type natriuretic peptide; RR: risk ratio; sST2: soluble interleukin-like protein receptor.

We developed a mathematical model to assess risk of unfortunate outcome faced by CHF patients. The pattern observed was described by the following logistic regression equation:

$$\begin{array}{l} \text{P}=\frac{1}{{\text{1}+\text{e}}^{-\text{z}}}\times \text{100\%}\\ \text{z}=-\text{4.13}+{\text{X}}_{\text{P}}\times \text{1.13}+{\text{X}}_{\text{AF}}\times \text{1.14}+{\text{X}}_{\text{AMI}}\times \text{1.45}\\ \;\;+{\text{X}}_{\text{Anemia}}\times \text{1.16}+{\text{X}}_{\text{NT-proBNP}}\times \text{0.000515} \end{array}$$

where, P is the risk of lethal outcome being observed, e is a mathematical constant approximately equal to 2.71828, X_P_ = 1 if the patient had community-acquired pneumonia when included in the study, and = 0 if he/she had none, X_NT-proBNP_ is NT-proBNP level in pg/mL, X_AF_ = 1 if the patient has any form of atrial fibrillation and = 0 with sinus rhythm, X_AMI_ = 1 if the patient has previously suffered a myocardial infarction and = 0 if he/she has had none, Х_Anemia_ = 1 if hemoglobin level is below normal, and = 0 with normal hemoglobin values (Note: the normal hemoglobin values are ≥ 120 g/L for females and ≥ 130 g/L for males).

We termed the model obtained PIFA-N (an acronym for the poor prognosis factors we identified: Pneumonia, Infarction, atrial Fibrillation, Anemia, NT-proBNP).

The threshold value of the P logistic function was 23%, with P ≥ 23% heralding a high and P < 23%, low probability of lethal outcome.

**Figure 1:**
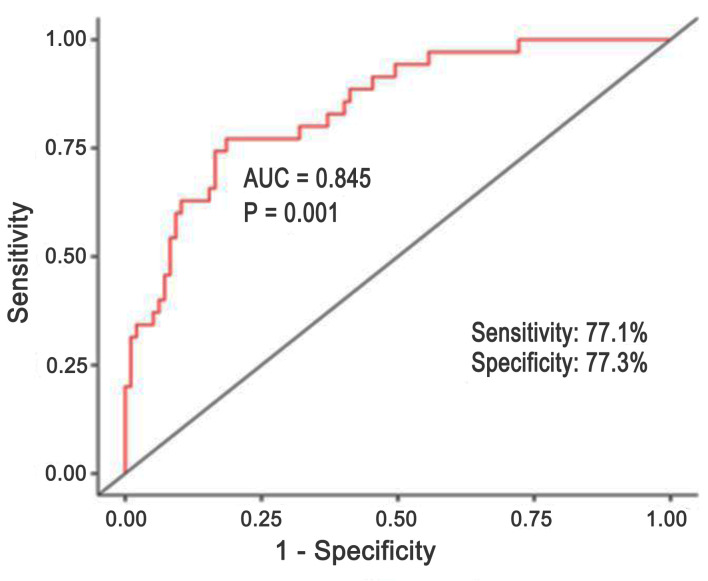
Results of ROC analysis for PIFA-N multivariate logistic regression model. ROC: receiver operating characteristic.

We performed ROC analysis (Fig. 1) to assess our developed model’s sensitivity and specificity.

Our model’s AUC was 0.845. The model we developed is 77.1% sensitive, 77.3% specific, and 77.3% diagnostically efficient.

## Discussion

**Figure 2:**
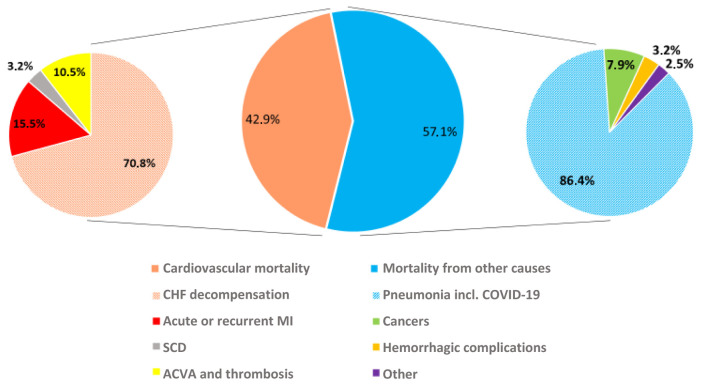
Cardiovascular and non-cardiovascular deaths of chronic heart failure (CHF) patients.

Our study demonstrated a high rate of death from all causes, with non-cardiac deaths prevailing, among CHF patients, which might be attributable to a serious-case and comorbid patient mix under study and also to special conditions prevailing in our prospective observation period, that coincided with the SARS-CoV-2 pandemic, and suboptimal therapy received during the observation period. Thus, events associated with SARS-CoV2 infection accounted for 86% non-cardiac deaths (Fig. 2).

According to the findings of the 33-year Framingham Heart Study, up to 33% of female and 22% of male CHFrEF patients’ deaths are not directly related to cardiovascular diseases, while among CHFpEF patients, non-cardiac causes account for up to 50% of deaths [11]. According to Jones et al’ meta-analysis, CHF patients’ risk of dying within 1 year may vary between 6% and 77% [12], with significant differences among regions.

By now, a number of scales for assessing CHF patients’ prognosis, namely MAGGIC-HF, SHFM, PRIDICT-HF, and BCN-Bio-HF, have been developed and validated on US and European populations of CHF patients. On comparative analysis, none of the scales showed a visible advantage over the rest, yet inclusion of NT-proBNP in the analysis helped increase their prognostic significance [9]. For patients with acutely decompensated CHF, all the scales’ predictive value proved unsatisfactory [10]. That necessitates the development of new tools for assessing patients’ prognosis in order to identify high risk groups.

In our study, as we developed our model, we used the results of univariate regression analysis to identify a number of anamnestic and clinical factors and biomarkers that showed their own predictive significance, including the levels of NT-proBNP and ST2. After multivariate analysis, some of the variables were not included in the finalized model.

The arguments used in the PIFA-N mathematical model we developed are easily verifiable conditions and routine laboratory figures that can be obtained without special or costly examination techniques: presence of a history of myocardial infarction (OR: 4.26, 95% CI 1.54–11.7, P = 0.005) and any form of atrial fibrillation (OR: 3.13, 95% CI 1.05–9.2, P = 0.039), community-acquired pneumonia (OR: 3.09, 95% CI 1.13–8.5, P = 0.028) and anemia (OR: 3.18, 95% CI 1.003–10.1, P = 0.049) on inclusion in the study, and NT-proBNP level in pg/mL (OR: 1.0005, 95% CI 1.0002–1.0008, P = 0.002). That is, we showed a significant negative influence of cardiac and non-cardiac comorbid conditions on prognosis. Similar data on the effect of comorbidities on CHF patients’ prognoses are cited by Paolillo et al [13].

We found such potentially important factors as age, male gender, body mass index, GFR or any echocardiographic parameters to have no significant influence on patients’ survival rate.

Notably, our model’s AUC is 0.845, which is comparable to other validated calculators such as the Seattle model (SHFM) [9].

The structure of the model we obtained is generally similar to the calculator developed by Barge-Caballero et al (2022) [14] and including five points: four clinical factors and one biomarker, namely NYHA FC III-IV, signs of congestion, hospitalization for HF decompensation in the preceding year, daily dose of Furosemide or analog ≥ 40 mg, and NT-proBNP ≥ 1,000 pg/mL. Unlike ours, Barge-Caballero’s model overlooked CHF etiology factors or comorbidities. Our model emphasized the negative predictive significance of ischemic nature of CHF, especially a history of myocardial infarction. According to Karoli et al, a history of acute myocardial infarction (AMI) increased the CHF decompensation risk by a factor of 3.6 [15]. In our model, a previous infarction is an even more important contributor (OR: 4.26, 95% CI 1.54–11.7, P = 0.005).

Tellingly, among a broad spectrum of biomarkers studied, only NT-proBNP showed its predictive significance. This matches the conclusion by Codina et al that the inclusion of NT-proBNP value in any prognostic model improves the latter’s predictive capability [9]. Also important is the fact that in our PIFA-N scale, like in the PREDICT-HF and BCN-Bio-HF calculators, the NT-proBNP level is a continuous rather than dichotomous variable [10]. Our prognosis assessment formula incorporates the level of NT-proBNP, which may change due to therapy, and anemia, a potentially curable condition; therefore, our proposed model does not assess the risks of adverse outcome as some constant quantity.

We considered various biochemical markers, namely sST2, galectin-3, haptoglobin, hepcidin, and copeptin, as possible variables that might increase our model’s predictive significance. However, our study did not confirm that hypothesis. The findings we obtained are in line with the worldwide trends [16]. Comprehensive assessment of the involvement of various neurohumoral systems in CHF pathogenesis and measuring the concentrations of various biomarkers as potential prognostic markers have repeatedly been attempted in the last few years [17, 18]. Those studies arrived at mixed findings, so by now the ESC and ACC/American Heart Association (АНА) CHF treatment guidelines only include NT-proBNP [16, 19, 20].

According to a Scientific Statement by the AHA, a combination of biomarkers may be more informative in the assessment of adverse outcome risks than individual biomarkers [17]. In most multi-marker models, new biologically active molecules are added to previously known risk factors, both clinical and laboratory ones [20]. The number of biomarkers being studied is usually within two or three, while the Bio-SHIFT prospective study contains a dynamic assessment of 92 biomarkers, including proteomic ones [19]. However, only NT-proBNP has come into broad practical use, and the question of what combination of biomarkers is the most predictively efficient remains open and calls for further research [16, 19, 20].

Interestingly, our PIFA-N model shares some components with the widely used CHA_2_DS_2_-VASc score, recently shown to be predictive of mortality among heart failure patients irrespective of whether they have atrial fibrillation [21, 22]. On the other hand, in our PIFA-N model, atrial fibrillation serves as an independent predictor of adverse prognosis. In addition, the NT-proBNP level used in PIFA-N makes it possible to assess the pathogenetic basis of CHF advancement. Comparative analysis of the incremental prognostic value of the PIFA-N and CHA_2_DS_2_-VASc models in our cohort may become the object of future research.

A critical review of literature and our own observations suggest that the concept of a multifactor (multi-marker) approach to prognosis assessment is not always realized based on various combination of new biomarkers [6, 9, 14, 16, 17]. In the case of our study, we implemented a multi-marker approach and a multifactor model by combining simple and accessible clinical and anamnestic variables with the “universal” CHF biomarker, NT-proBNP.

### Conclusion

We analyzed CHF patients’ 3-year survival rate and developed PIFA-N, an efficient prognostic model for assessing the risk of lethal outcome, that factors in myocardial infarction, atrial fibrillation, community-acquired pneumonia, anemia on inclusion in the study, and NT-proBNP level.

## Data Availability

The data supporting the findings of this study are available from the corresponding author upon reasonable request.
